# Beyond the barrier: the immune-inspired pathways of tumor extravasation

**DOI:** 10.1186/s12964-023-01429-1

**Published:** 2024-02-08

**Authors:** Sara Di Russo, Francesca Romana Liberati, Agnese Riva, Federica Di Fonzo, Alberto Macone, Giorgio Giardina, Marzia Arese, Serena Rinaldo, Francesca Cutruzzolà, Alessio Paone

**Affiliations:** Department of Biochemical Sciences “Alessandro Rossi Fanelli”, Sapienza University of Rome, Laboratory affiliated to Istituto Pasteur Italia-Fondazione Cenci Bolognetti P.Le A. Moro 5, Rome, 00185 Italy

**Keywords:** Extravasation, Metastasis, Endothelial cells, Immune surveillance, Tumor-immune associations, Neutrophils, Inflammatory mediators, Endothelium alteration, Immune cell mimicry, Tumor cell infiltration

## Abstract

Extravasation is a fundamental step in the metastatic journey, where cancer cells exit the bloodstream and breach the endothelial cell barrier to infiltrate target tissues. The tactics cancer cells employ are sophisticated, closely reflecting those used by the immune system for tissue surveillance. Remarkably, tumor cells have been observed to form distinct associations or clusters with immune cells where neutrophils stand out as particularly crucial partners. These interactions are not accidental; they are critical for cancer cells to exploit the immune functions of neutrophils and successfully extravasate. In another strategy, tumor cells mimic the behavior and characteristics of immune cells. They release a suite of inflammatory mediators, which under normal circumstances, guide the processes of endothelium reshaping and facilitate the entry and movement of immune cells within tissues. In this review, we offer a new perspective on the tactics employed by cancer cells to extravasate and infiltrate target tissues. We delve into the myriad mechanisms that tumor cells borrow, adapt, and refine from the immune playbook.

Video Abstract

**Supplementary Information:**

The online version contains supplementary material available at 10.1186/s12964-023-01429-1.

## Introduction

**Fig. 1:**
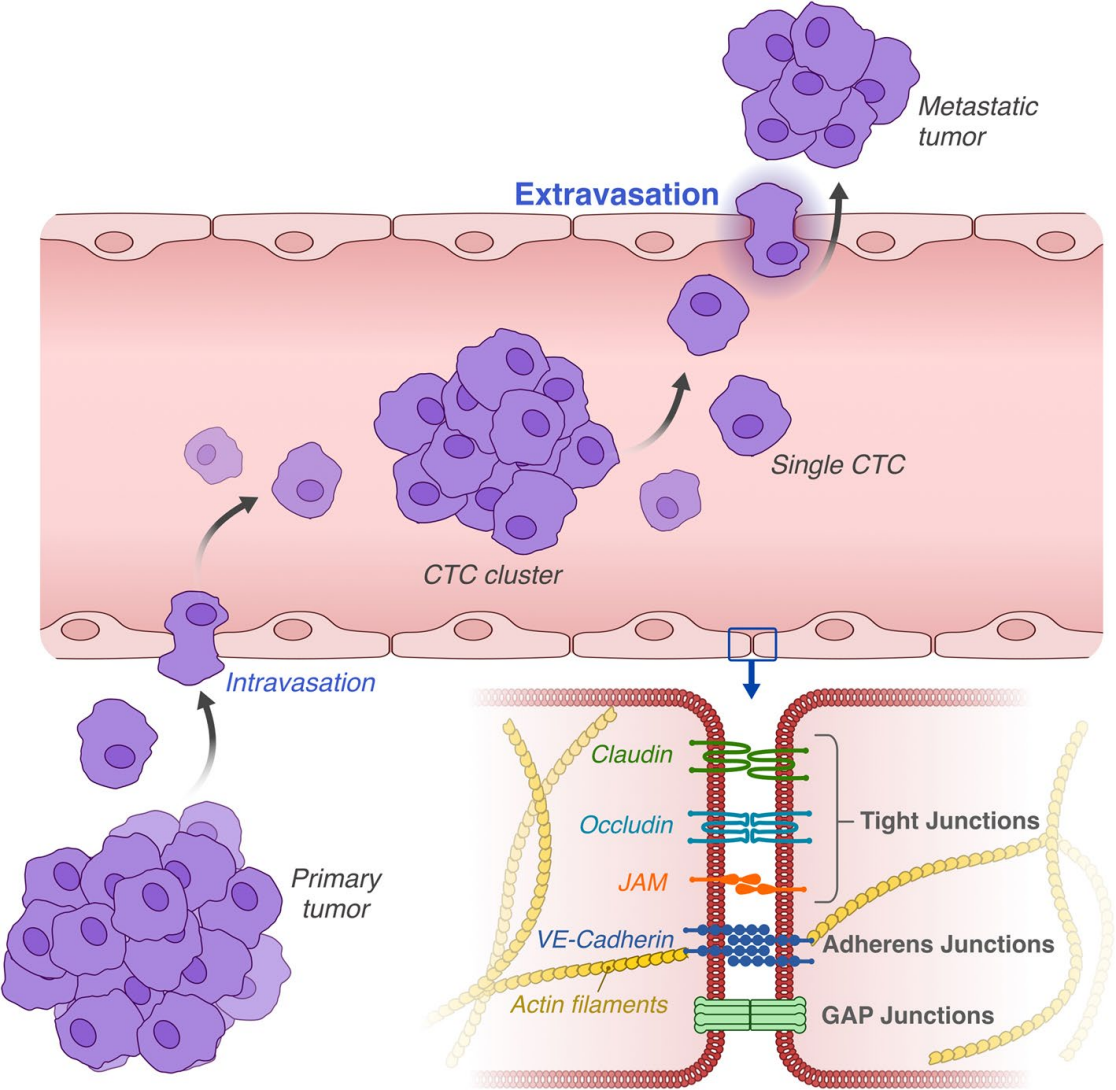
Overview of the metastatic process. At the bottom, a detailed depiction of the key molecules involved in the junction mechanisms between endothelial cells

The metastatic process, while seemingly straightforward in concept, is in reality a complex and elusive phenomenon. Metastasis begins when a tumor cell detaches from the primary tumor. The cell manages to detach from the basal lamina, to which it’s typically anchored, and embarks on an intricate journey, becoming circulating tumor cells (CTCs). It first enters the bloodstream via a process known as intravasation. It then utilizes either the vascular network or the lymphatic system to circulate throughout the body. In this review, however, we will focus specifically on the vascular route of CTCs. The detailed processes of intravasation and the circulation of cancer cells through the lymphatic system will not be covered. Once the CTC reaches its target organ through the vascular pathway, it undergoes extravasation. This critical step involves the cell invading the target organ, where it can proliferate and develop into a full-blown metastasis (Fig. 1) [1].

Despite Stephen Paget’s groundbreaking theory in 1889, which likened metastatic cells to ‘seeds’ that spread throughout the body, but only ‘take root’ in certain organs, the mechanisms dictating this organ specificity remain largely unclear even today [2]. This theory has been compared to two other hypotheses that followed: i) Ewing’s “Blood Flux” or “Anatomical and Mechanical” Theory from 1928 suggested that metastatic cells simply followed the flow of blood to target organs, influenced by physical proximity and the vascular layout of the organ [3] and ii) the Chemotropism Theory, proposed that specific organs release chemical substances that act like magnets for tumor cells. The metastatic process is probably shaped by a blend of these factors and possibly others, indicating the complexity of how cancer cells choose their target organs [4]. Recent research often refers to the concept of the “pre-metastatic niche”, underscoring the idea that primary tumors can precondition distant sites for the arrival and implantation of cancer cells [5]. This comprehensive concept amalgamates elements of all three aforementioned theories, enhanced by our current understanding of the molecular biology of cancer and the role the immune system plays in cancer progression.

### CTCs

CTCs are defined as tumor cells that leave the primary tumor and enter the bloodstream or lymphatic system to lead to the formation of metastases. Recent transcriptomic and proteomic studies using single-cell analysis have confirmed the idea that CTC populations from individual patients are highly heterogeneous, with only a subset of cells likely possessing the specific characteristics necessary to succeed in various steps of the metastatic process and to form metastases in specific target organs [6]. Despite the considerable variability seen within populations of metastatic cells, researchers have identified some consistent characteristics that are valuable for isolating CTCs. These findings also hold promise for improving prognostic assessments [7]. The majority of CTCs primarily originate from epithelial tissues. A specific process allows these cells to acquire a distinct phenotype typical of mesenchymal cells when necessary and then revert to a more epithelial phenotype once they reach their target organ. This remarkable ability is known as “epithelial to mesenchymal plasticity”. The initial transition towards a more mesenchymal phenotype equips these cells with a set of characteristics associated with metastatic behavior, such as the ability to migrate, invade, and survive when not adhered to other cells or surfaces. This phase is commonly referred to as “epithelial to mesenchymal transition” (EMT). Conversely, the reverse process, in which the cells return to an epithelial phenotype, is called “mesenchymal to epithelial transition” (MET). MET is considered a crucial phenomenon in the later stages of the metastatic process [8]. One common characteristic often observed in many CTCs is the expression of the epithelial marker EpCAM, whose function is not yet fully understood but appears to influence various aspects of CTCs behavior [9]. However, it is important to note that not all types of primary tumors will give rise to CTCs expressing EpCAM. Nonetheless, EpCAM remains one of the most commonly used markers, serving as a negative prognostic factor and facilitating the isolation of CTCs from the blood when combined with the absence of CD45, a typical leukocyte marker [10]. For specific tumor types that do not seem to produce EpCAM ^+^ CD45^-^ cells, the classification based on high CD44 and low CD24 expression (CD44 ^+^ /CD24 ^− /low^) has been introduced. This classification appears to be associated with a subset of CTCs that exhibit characteristics typical of cancer stem cells (CSCs) [9, 11]. It is frequently employed in breast cancer, and breast tumors displaying this expression pattern tend to demonstrate enhanced invasion and metastasis [11].

### Extravasation process

Extravasation represents a bottleneck in the metastatic process. Despite a large number of cells leaving the primary tumor, only a few ultimately succeed in forming full-blown metastases. Therefore, understanding the processes governing extravasation is crucial for identifying effective targets to inhibit the entire metastatic process. Tumor cells encounter at least three significant hurdles during or immediately after the process of extravasation: 1) Adhesion: CTC must first adhere to and then cross a barrier formed by endothelial cells. 2) Immune Surveillance: during their journey in the bloodstream, CTCs are particularly vulnerable to attacks from immune cells, such as patrolling nonclassical monocytes [12]. They must therefore find a way to survive these additional assaults. 3) Adaptation to a foreign microenvironment: once inside the parenchyma of the target organ, tumor cells find themselves in a completely alien microenvironment, both metabolically and in terms of growth signals. The cells must adapt to this new microenvironment, utilizing new metabolic substrates, thus profoundly altering their metabolic machinery [13]. They must also acquire growth signals that support the proliferation, which are likely different from those utilized within the primary tumor. This stage is particularly critical, and the process itself will only select the few cells capable of surviving under these “extreme” conditions. This selection, in turn, produces cells that are particularly “aggressive”, precisely because they are capable of thriving under such harsh conditions.

### Endothelial cells

In capillary venules, where significant additional barriers to extravasation are absent, pericytes are sparser, and muscle cells and thick basal membranes are lacking, the primary barrier consists mainly of endothelial cells. To facilitate extravasation, tumor cells must engage in a complex and close interaction with the endothelium. Under certain conditions, endothelial cells often lose their barrier function and transform, morphing into a gateway that allows tumor cells to enter the target tissue [14]. Endothelial cells line the inner surface of blood vessels, separating the vessel lumen from the tissue through which it passes. To properly fulfill their function, these cells must form a solid, continuous layer intimately bound together. To achieve this effect, cells employ three distinct junction mechanisms: 1) Tight Junctions: these junctions link the membrane of one cell to another, particularly through proteins like claudins and occludins. These connections tightly bind cells to each other via individual filaments, creating a barrier that is virtually impermeable to the passage of cells and molecules [15, 16]. 2) Adherens Junctions: these junctions establish a robust bond between cells. They are composed of proteins such as cadherins, which interact with the cell’s cytoskeleton, providing structural solidity to the junction, even under conditions of substantial mechanical stress [17]. 3) Gap Junctions: primarily formed by connexins, these junctions do not directly participate in the structure of the vessel wall (Fig. 1) [18]. Instead, they create pores that allow the intercellular exchange of molecules. Collectively, these protein complexes form a moderately selective barrier, controlling the entry and exit of molecules and cells from the bloodstream under specific conditions.

### Extravasation mechanisms

The extravasation process was initially characterized and studied in immune system cells, although many details governing the extravasation of tumor cells overlap with those of immunity. In fact, tumor cells seem to mimic or harness the immune system’s mechanisms to gain access to tissues, an ability that should ideally be exclusive to the immune system. Initially, CTCs navigate in the center of the blood vessels. In the first phase, these cells deviate from the main bloodstream, moving towards the periphery of the flux known as the ‘fringe’ via a process termed ‘margination’. Here, cells gravitate towards the margins of the blood flow, where the pressure is lower and thus the flow speed is significantly reduced [19]. Subsequently, CTCs establish contact with the endothelial cells lining the blood vessels. This leads into the second phase, known as ‘rolling’, characterized by a deceleration of these cells. Following this deceleration, cells come to a complete halt before they undertake the third and final act of extravasation [20]. Each of these processes is facilitated by specific receptors. For instance, the ‘rolling’ process is mediated by selectins, a family of adhesion molecules expressed by both endothelial cells and immune cells [20]. Key selectins, such as L, P, and E selectins, are used by leukocytes during the rolling phase. Although this overall process bears a resemblance across cells, tumor cells do not necessarily employ the exact same molecules as leukocytes for extravasation [21]. In certain cases, this could partly account for the organ-specific tropism observed in particular types of tumors, which often migrate preferentially to specific target organs [22, 23]. Understanding these distinct patterns provides crucial insights into the control of immune responses and the spread of metastatic cancer cells.

Immune system cells can extravasate using two distinct mechanisms. Transcellular extravasation occurs when a leukocyte passes straight through the body (cytoplasm) of an endothelial cell. Following initial anchoring, the leukocyte clings to the endothelial cell and forms a ‘podosome’ [24, 25]. The leukocyte then exploits this podosome to establish a direct passage through the endothelial cell, including the reorganization of the endothelial cell’s actin cytoskeleton and the participation of different molecules such as ICAM-1 and PECAM-1 [15]. Paracellular extravasation occurs when a leukocyte moves across neighboring endothelial cells, taking advantage of intercellular gaps producing fenestrations between endothelial cells initially tightly bound together [21]. Generally, the increase in permeability is a transient phenomenon linked to the specific inflammatory stimulus and resolves relatively quickly. However, in the context of chronic inflammation or signals originating from tumors, this phenomenon can persist over time [26]. Physiologically, immune cells tend to extravasate through venules, which, in comparison to arterioles, display a higher permeability [27]. The endothelial cells in venules are generally “leakier” to fluids and solutes, and particularly, they are more susceptible to the control of mediators that can increase permeability. In most organs, leukocytes extravasate from venules exposed to the action of inflammatory cytokines, both through the transcellular route (which requires a thorough understanding) and through intercellular junctions [28]. To allow the leukocyte to move through, the tight and adherens junctions that normally hold these cells together must be partly and temporarily destroyed. Various proteins, including those from the JAM (junctional adhesion molecule) family, PECAM-1 and others, control the process [17, 21]. This route is closely managed to prevent plasma and other blood components from leaking into the surrounding tissues. Although transcellular route was previously assumed to be less prevalent, new research suggests that depending on the tissue and inflammatory setting, this mechanism of extravasation may be as common as or even more common than the paracellular route. Both extravasation methods physiologically allow immune cells to migrate to areas of infection or damage, but they also play roles in a variety of diseases, including cancer metastasis.

### Cancer cells extravasation

Cancer cells from various tumor origins appear to co-opt strategies traditionally linked to the immune system in order to invade tissue parenchyma. We have identified two main tactics, either directly involving or emulating the immune system, that CTCs can utilize for extravasation:

1. CTCs might collaborate with immune cells, forming integrated clusters and capitalizing on the innate abilities of these cells to facilitate their migration.
2. CTCs might emulate immune cells, assimilating characteristics that naturally enable the immune system to penetrate and occupy an organ.
3. CTCs might fuse with immune cells, acquiring some of their characteristics that could beneficial for efficient extravasation. These maneuvers underscore the sophistication and resilience of cancer cells in their persistent drive to invade and multiply.

### CTCs/neutrophils clusters

Neutrophils are a subset of polymorphonuclear cells that serve as the first responders to a site where an inflammatory signal is produced. This makes them the initial line of defense against infections. Regarding the metastatic potential of CTCs, neutrophils have been shown to exhibit dual and contrasting roles, acting as either pro-tumor or anti-tumor entities. The role they assume depends on the context, model, and most importantly, the type of “signal” produced by the tumor cells. While most CTCs are found as individual cells in the bloodstream, some group together to form clusters. These clusters can vary in their cellular homogeneity. Clustering, especially when cancer cells associate with immune cells, seems to provide an advantage, as it is significantly linked to a worse progression-free survival [29]. In most breast cancer patients, for example, CTCs circulate as isolated entities, 7.6% move associated in tumor-only clusters, and 3.4% circulate as clusters in association with neutrophils. Despite the fact that in 25% of CTC/white blood cell (WBC) clusters the immune cells are lymphocytes, the function of these cells remains largely elusive. On the other hand, neutrophils represent the cell population most commonly found in clusters with CTCs (85.5%-91,7%) [29]. Patients in whom neutrophil-associated clusters are present show lower rates of progression-free survival [29]. Neutrophil-associated clusters have been also reported in different mouse models. Interestingly, injecting in mice a specific number of tumor cells from either tumor-only clusters or tumor cells with associated neutrophils results in decreased survival rates for the latter group. This indicates that the association with neutrophils can modify the behavior of the tumor cells, potentially conferring a more aggressive phenotype.

**Fig. 2:**
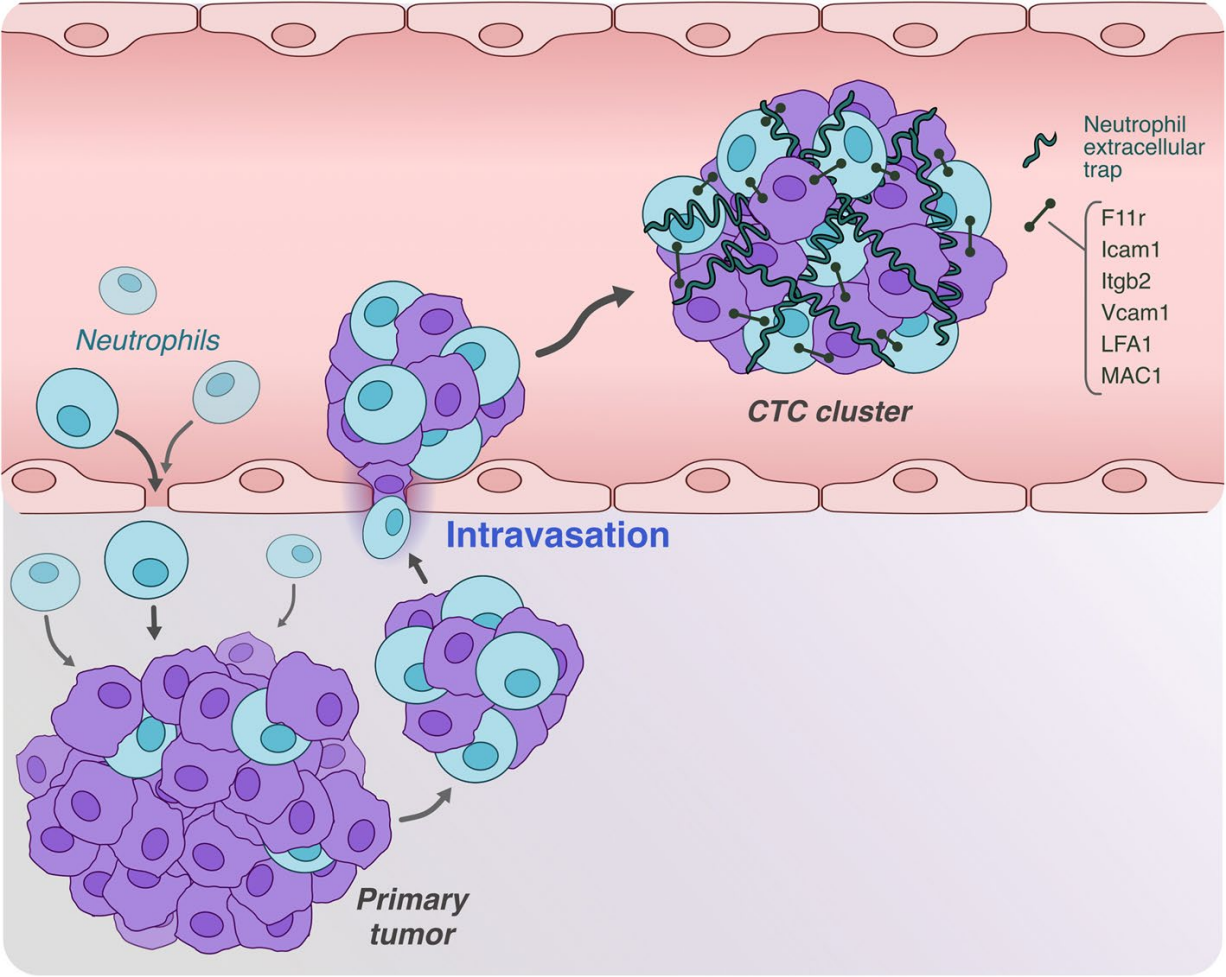
Mechanism of formation for tumor cell-neutrophil cluster: focus on the principal molecules involved

It is very fundamental to note that the association with tumor cells tends to occur at the site of the primary tumor (Fig. 2).

Inhibition of infiltration of the primary tumor by neutrophils with antibodies blocking the neutrophil surface antigen Ly-6 g, completely eliminates the formation of mixed tumor/neutrophil clusters. On the contrary, stimulation of neutrophils with intratumoral injections of G-CSF, a stimulating factor, leads to increased infiltration of these cells into the tumor, boosting the number of circulating tumor cell/neutrophil complexes, and reducing the survival rates of the treated mice.

Subsequent parallel analyses of RNA and DNA sequencing revealed that a mutation of the TLE1 gene is commonly observed in many patients with neutrophil clusters in their blood [29]. TLE1 is part of the co-repressor family known as the transducin-like enhancer of split, which does not directly bind to DNA but instead helps to form a complex that blocks the activity of specific transcription factors. TLE1 is specifically a transcriptional repressor involved in controlling inflammatory phenomena. A study on a mouse model demonstrated that inactivating mutations in this gene lead to an increase in NFκB activity, which results in a heightened response to inflammatory stimuli and the development of inflammation-associated tumors [29–31]. Single-cell transcriptomic studies suggest that CTCs originating from clusters with neutrophils exhibit an upregulation in several genes associated with cell proliferation, likely promoting cellular growth. The interaction between these two cell types seems to be primarily driven by the cytokines IL-6 and IL-1b, along with their respective receptors. Additionally, the G-CSF factor secreted by CTCs plays a pivotal role in this relationship [32]. Cells organized in clusters seem to form what has been proposed as a “portable niche”, where the cells form a kind of synapse that allows a spatially controlled and thus highly effective exchange of factors [33]. In particular, it is hypothesized that the TLE1 mutations observed in patients with high CTC/neutrophil clusters are due to an increased activity of NFκB, which induces the production of G-CSF in the primary tumor. This in turn would recruit granulocytes, initiating the formation of the clusters [30].

### Clusters formation mechanism (Fig. 2)

In addition to the previously mentioned increase in cell cycle-related genes, it appears that CTC / neutrophil clusters show an increase in various cell–cell adhesion molecules such as F11r, ICAM-1, Itgb2, and VCAM-1. Subsequent knockout analyses identified VCAM-1 as the primary contributor to the formation of these clusters [29]. In a melanoma model, it was demonstrated that while Lymphocyte function-associated molecule-1 (LFA-1) is involved in the initial capture of neutrophils, the interaction between ICAM-1 expressed on tumor cells and Mac-1 expressed on neutrophils is, on the other hand, more involved in maintaining clusters, particularly when stimulated with inflammatory cytokines [34]. In their physiological function, neutrophils can form structures akin to a net, termed Neutrophil Extracellular Traps (NETs), which serve to ensnare pathogens for easy destruction. During interaction with cancer cells, neutrophils seem to engulf CTCs within the NET, facilitating the metastatic process. This theory is supported by the observation that metastasis formation is reduced by treatment with DNase and Elastase, which are known to induce NET degradation. An involvement of B1 integrin, produced in the CTCs/NET interaction process, was also observed. Specifically, B1 integrin is produced by both CTCs and neutrophils and is exposed on the NETs, and its interaction determines the binding of the CTCs to the complex. Furthermore, it was observed that inflammatory events induce an increase of B1 integrin in the NETs [35].

### Neutrophils and their role in cancer cell extravasation

As previously mentioned, the process of extravasation initiates with a rolling action of cells, followed by their adherence to the endothelial lining, before they infiltrate the parenchyma of the targeted tissue. Recent research has unveiled that neutrophils can enhance the interaction between endothelial cells and cancerous ones. While the strategies adopted may vary according to different models and experimental environments, neutrophils seem to leverage their immune cell functions to assist tumor cells in the extravasation process.

**Fig. 3:**
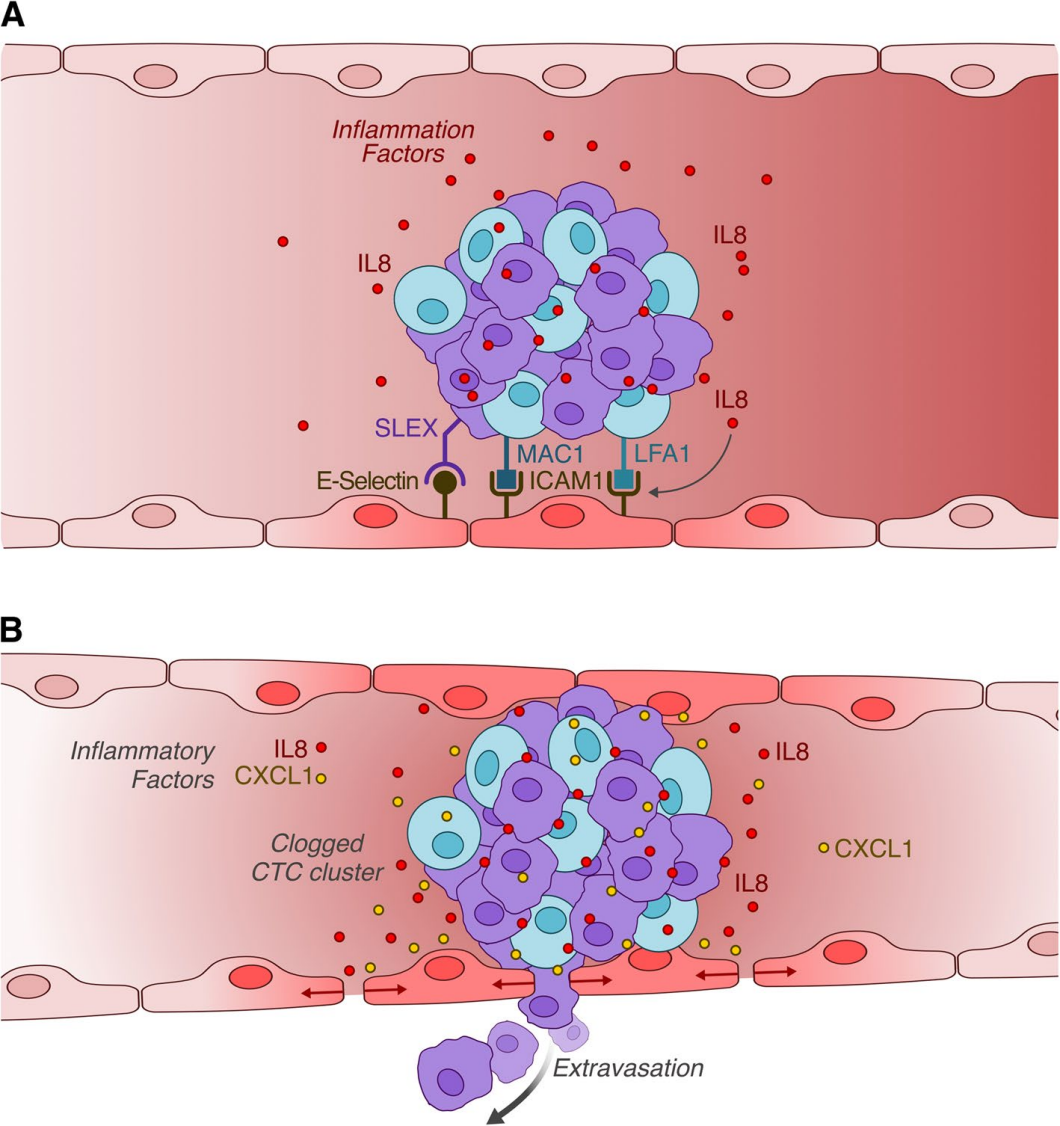
Advantages of tumor cell clusters containing neutrophils: **A** Mechanisms of adhesion to the endothelium. **B** Mechanisms promoting endothelial barrier disruption, facilitating extravasation

Neutrophils appear to effectively slow down the entire cellular cluster by establishing direct interactions with endothelial cells. This serves as an anchoring mechanism, facilitating a rolling effect that enables more controlled movement of the entire cell group. In this mechanism, beta (2)-integrins like MAC-1 and LFA-1, which are typically produced and employed by neutrophils during extravasation, bind to ICAM-1 expressed on the endothelium. Experiments conducted under flow conditions have clarified that, as previously hinted regarding the cluster formation mechanism between CTCs and neutrophils, LFA-1 is crucial in the initial stages of interaction with the endothelium. The stabilization of these interactions, on the other hand, is entrusted to MAC-1 [34]. Both these molecules can potentially be modulated by inflammation [34, 36]. In the context of a melanoma model, for instance, IL-8 produced by CTCs attracts neutrophils. This then promotes the adhesion of CTC/neutrophil clusters to the endothelium by inducing both MAC-1 and ICAM-1 [36]. Furthermore, in an in vivo liver tumor model, it was demonstrated that neutrophils expedite the adhesion of CTCs to the liver sinusoidal endothelium. This occurs through a mechanism mediated by E-selectin ligands containing Sialyl-Lewis X (SLEX) fractions that are expressed on the tumor cells’ surface, and by E-selectin expressed by endothelial cells (Fig. 3A) [37]. In addition to facilitating adherence processes with the endothelium, neutrophils also mediate the actual extravasation process, largely through their primary functions as immune cells. To clarify this, a multiplexed microfluidic model of the human microvasculature was developed to observe the interactions between tumor cells and neutrophils [38]. Both cluster-bound and free neutrophils exhibit high migratory activity, although the cluster-bound neutrophils move at a slower pace. Interestingly, the neutrophils never detach from the cluster but remain confined to the area surrounding it. Both CXCL1, produced by tumor cells, and Interleukin-8 (IL-8), produced by neutrophils, play critical roles in restricting the neutrophils to the vicinity of the cluster. This restriction, known as chemotactic confinement, is especially crucial for the extravasation of the cluster’s tumor cells. Tumor cells in clusters with neutrophils migrate more efficiently compared to tumor-only cell clusters. The migration process of the former is halted in the presence of IL-8 blocking antibodies. This is probably because IL-8 is known to influence the permeability of the endothelium, thereby facilitating the extravasation process. The accumulation of neutrophils at tumor sites likely enables IL-8 to concentrate and exert its effects on the nearby endothelial cells efficiently [39]. In various murine breast cancer models, a comparable mechanism is mediated by neutrophils’ secretion of IL-1B and matrix metalloproteinases [40]. This supports the concept that while the molecules mediating the process might differ, the phenomenon is invariably mediated by inflammatory molecules released by neutrophils. In this specific context, neutrophils provide a solution for a type of cells incapable of otherwise producing inflammatory factors, affirming their vital role in the process and the higher efficiency in the extravasation of mixed clusters (Fig. 3B).

### Mimicking immune cells central role of NF-κB (Fig. 4)

Immune cells efficiently perform their functions primarily through interactions with other cells of the immune system. This interaction predominantly happens through an exchange of chemical messengers, the inflammatory factors, each with distinct and specific roles. These messengers can be released when specific cells come into contact with a pathogen or a fragment of it, or upon receiving a similar signal, which triggers an amplification mechanism. This process is permitted by the activation of a specific signal transduction pathway. Although various signaling routes, such as JAK/STAT and MAPK, have been identified as regulators of inflammatory mediator production or release in certain models, the full scope and significance of their involvement are still not completely clear [41, 42]. In contrast, this review will focus on the NF-κB pathway, widely considered the central regulator orchestrating the expression of genes associated with inflammatory responses. The ability to activate the NF-κB pathway isn’t exclusive to the immune system; various other cells also possess pathogen recognition tools and can trigger it to initiate an immune response. This implies that nearly all cells within our body have the inherent potential to start an inflammatory response. It’s widely recognized that many cancer cells constitutively display elevated levels of NF-κB activation. As a result, there’s a continuous production of inflammatory mediators that assume multiple roles favoring tumor growth. This phenomenon provides a tactical edge for tumor cells, allowing them to mimic activated immune cells. The inflammatory factors they produce essentially act as a gateway for them to infiltrate tissues, mirroring the capabilities of the immune system [43].

**Fig. 4:**
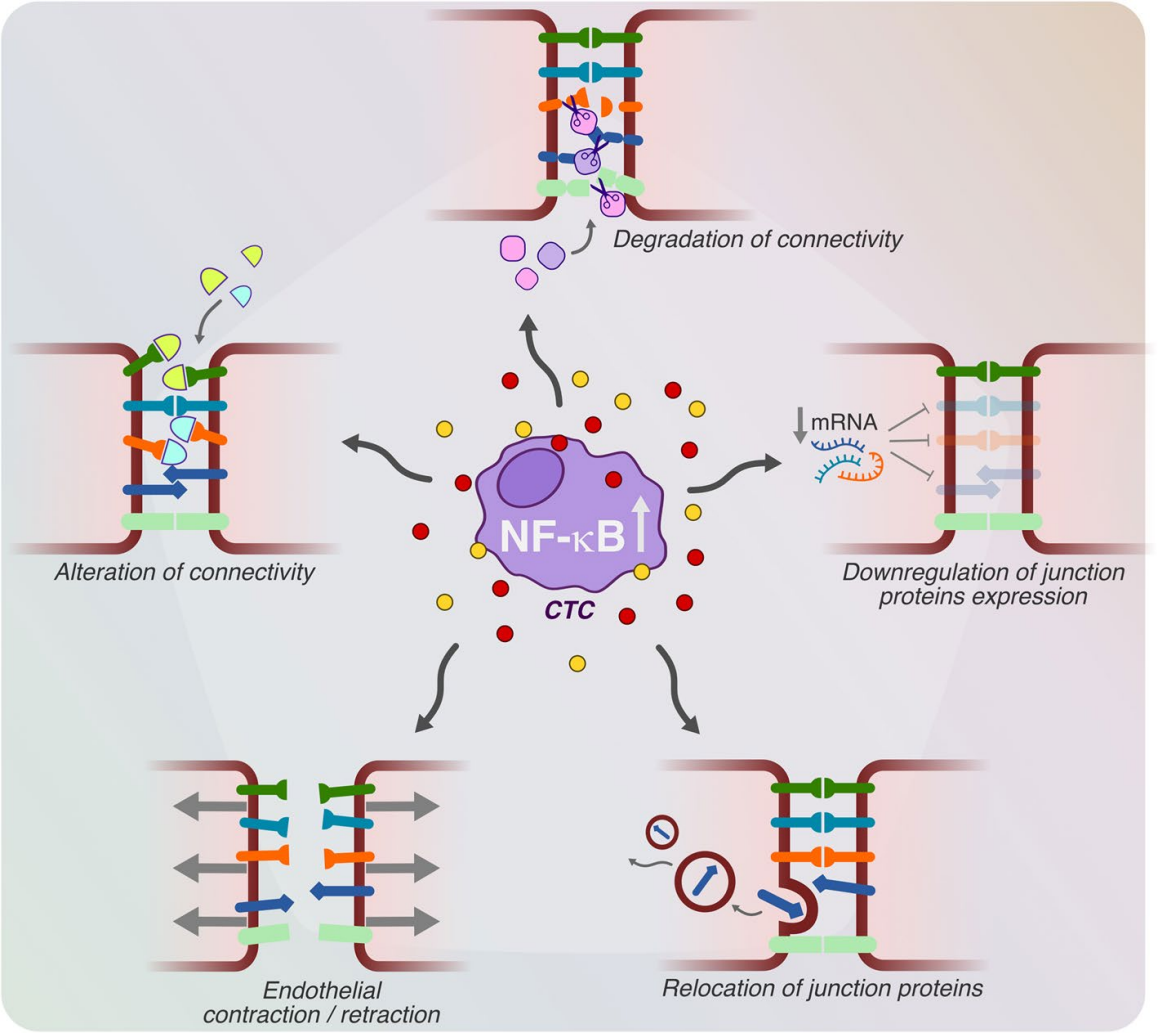
Effect of secreted factors on the junctions between endothelial cells. Key mechanisms by which factors secreted from immune /tumor cells increase the permeability of the endothelial barrier to cell passage

The NFκB pathway, which is finely balanced by inducers and inhibitors, plays a crucial role in tumorigenesis. This role was established several years ago with the discovery of the v-Rel oncogene from the avian Rev-T retrovirus, an analog of c-Rel—a key subunit of NF-κB found in humans [44]. Typically, mutations that cause constitutive activation of the NF-κB signal are primarily found in molecules at the beginning of the pathway. For instance, in diffuse large B-cell lymphoma (DLBCL) and multiple myeloma (MM), proteins such as CD79 and MyD88 trigger NFκB activation through gain-of-function mutations [45, 46]. Additionally, loss-of-function mutations in negative regulators like CYLD, A20, and TRAF3 also lead to abnormal activation of NF-κB [47]. A well-characterized model is DLBCL, where mutations in three proteins—Rel, IκB, and p300—have been found to contribute to NFκB activation in the human B-cell lymphoma cell line RC-K8 [48]. While there are numerous mutations in genes involved in NFκB pathway, the number of tumors displaying hyperactivation of the pathway far exceeds those with observed mutations in any involved genes. In cases without apparent mutations, the activation of the pathway in tumor cells often stems from an increase in factors that induce activation of the pathway’s upstream receptors—such as cytokines like IL-6, TNFα, and IL-1—within the tumor microenvironment [49]. These factors can be released by the tumor itself, setting off an autocrine loop mechanism, or in some instances, may be released by other cells in the microenvironment stimulated by tumor-derived factors. For example, several microRNAs like miR21 and miR29a secreted by Lewis Lung Carcinoma cells (LLC) can activate immune system cells, particularly lung-resident macrophages, through specific toll-like receptors. This activation, in turn, triggers the secretion of cytokines such as IL-6 and TNFα, supporting the proliferation of tumor cells and formation of lung metastases [50]. Another critical factor to consider is the interconnectedness of the NFκB pathway with many others that can influence and regulate its activity. Many of these intersecting pathways play a role in tumor onset or progression. For example, the reduction in BRCA1 expression, often seen in various types of tumors, most notably breast cancer, has been demonstrated to increase phosphorylation of serine 536 of the canonical pathway effector p65 and processing of the p100/p52 complex, ultimately activating both the NF-κB canonical and non-canonical pathways thus promoting cell proliferation [51]. In a specific subset of breast cancer patients, the circular RNA circIκBκB is overexpressed and induces the creation of a pre-metastatic niche through the promotion of osteoclastogenesis, critical for bone metastasis formation. This mechanism is mediated by the degradation of the inhibitor IKBa through the activation of the inducer Iκκb, ultimately leading to the activation of NFκB that transcribes a group of genes involved in bone remodeling. This process is activated by the splicing factor EIF4A3, which promotes the cyclization of circIκBκB by binding to the regions flanking this RNA [52].

Genes responsible for initiating patterns of inflammatory factors production can be activated in two settings: either within immune cells associated with tumor cells or directly within the tumor cells themselves. The inflammatory factors thus released play a critical role in modifying the permeability of the endothelial barrier, thereby allowing entry into the target tissue. It could be hypothesized that the unique profile of activated inflammatory factors in different types of tumor cells or clusters, along with their interaction with other factors produced in various organs and tissues, may specifically facilitate increased permeability in a given organ. This, in turn, could contribute to the organ-specific targeting, or “organotropism,” observed in certain types of tumors. Although it’s generally agreed upon that the transendothelial migration is commonly used by cancer cells for crossing the endothelial barrier [53], the intricacies of this process still remain largely elusive. Intriguingly, preliminary evidence from in vitro studies indicates that the pro-inflammatory molecule TNFα may favor this migratory behavior [54]. The ensuing discussion specifically focuses on paracellular permeability, delving into the different mechanisms employed by tumor cells to increase the permeability of the endothelial barrier. Vascular structural stability is attributed to certain molecules like occludins, claudins, and VE-cadherin, which provide cohesion between endothelial cells. However, certain factors specifically target these structural molecules, undermining the integrity of the barrier as detailed in the following paragraph.

### Factors altering intercellular connectivity

Various factors can lead to the alteration of cell-to-cell contacts, effectively widening the space between endothelial cells and disrupting their unified structure. For instance, Fibrinogen and other factors like cAngptl4 and Ephrin A1 directly interfere with cellular junctions, creating a more permeable barrier. Specifically, epithelial tumor cells are known to produce elevated levels of angptl4. This molecule binds to integrin α5β1, claudin-5, and VE-cadherin on endothelial cells, subsequently undermining the integrity of cell-to-cell contacts. Moreover, the interaction between angptl4 and integrin α5β1 activates Rac1/PAK signaling pathways, thereby further weakening the cellular junctions [55].

In another example, CXCL5 activates the CXCR2 receptor and triggers p38 MAPK signaling, which in turn has been observed to increase permeability in a model of the blood–brain barrier [56]. Not surprisingly elevated levels of CXCL5 have been documented in various types of tumors, including cholangiocarcinoma and hepatocellular carcinoma [57].

Additionally, Ephrin-A1 undergoes cleavage by ADAM12 to produce a soluble form. This soluble variant is capable of binding to the EphA1/A2 receptor, thereby interfering with the already established Ephrin-EphA interactions between endothelial cells and it has been demonstrated to participate in the mechanism of metastasis formation in a lung cancer model [58, 59].

Finally, Fibrinogen has been found to attach to the extracellular domain of VE-cadherin. This binding event effectively disrupts the cell-to-cell interactions that are critical for the structural integrity of cellular barriers [60, 61]. Elevated levels of plasma Fibrinogen have been detected in multiple types of cancer, including those of the stomach, colon, and pancreas [62].

### Factors degrading junction proteins

Matrix metalloproteinases (MMPs) constitute a group of extracellular, zinc-reliant enzymes that have the collective ability to break down components of the extracellular matrix (ECM). Numerous studies indicate their significant involvement in various stages of cancer progression [63]. Certain metalloproteases, specifically ADAM10, ADAM17, and MMP3, play a pivotal role in increasing endothelial permeability by cleaving proteins that are crucial for maintaining cell-to-cell connections. For example, ADAM17 is responsible for the degradation of Junctional Adhesion Molecule A (JAM-A), disrupting the integrity of tight junctions between endothelial cells. Similarly, ADAM10 contributes to the dissolution of adherens junctions by targeting and breaking down VE-cadherin, a protein essential for cell adhesion. MMP3 appears to regulate the permeability of the blood–brain barrier (BBB) cells by modulating the abundance of tight junction (TJ) proteins as well as VE-cadherin. Through these mechanisms, these metalloproteases exert a substantial impact on endothelial integrity, influencing vascular permeability [64–66].

### Factors reducing junction protein expression

The downregulation of junction proteins, which is not yet fully understood, can occur through various mechanisms. For example, CXCL12, found in fibroblasts, but also overexpressed in many cancer types [67], interacts with the CXCR4 receptor, leading to a decrease in the levels of ZO-1, occludin, and VE-cadherin [68]. Hepatocyte growth factor, prevalent in breast cancer cells, targets the c-met receptor and influences ZO-1 phosphorylation, resulting in lower levels of this protein [69]. Similarly, although its role in tumor progression remains unclear, IFN gamma functions to reorganize and reduce the expression of VE-cadherin [70].

Physiologically produced by peripheral blood mononuclear cells (PBMCs) and macrophages, resistin is also overexpressed in various types of tumors. This factor interacts with the TLR4 receptor, initiating a signaling cascade that activates key molecules such as p38, NADPH oxidase, and CREB. This, in turn, leads to the reduced production of the tight junction proteins ZO-1 and occludin [71, 72]. Additionally, the proteins S100A4 and S100A8/A9, which are not only secreted by inflammatory cells but are also found to be overexpressed in various cancers, including lung, breast, and pancreatic cancers, as well as myeloid leukemia, play a role in the downregulation of the tight junction protein occludin [73]. Despite their structural similarities, these proteins interact with different receptors, since S100A8 connects with TLR4, while S100A9 binds to RAGE and induce the formation of actin stress fibers through pathways involving p38 and ERK phosphorylation that contribute to increase microvessel permeability (see below) [74, 75].

### Factors inducing structural reorganization of junction proteins

In some scenarios, targeted signaling events prompt a rearrangement of junctional proteins within cells. This generally involves the translocation of these proteins from their operational locations at the cell surface to different cellular compartments. This translocation subsequently leads to alterations in the local permeability of blood vessels. A classic example is the influence of VEGF/VEGFR2 signaling on VE-cadherin, which when phosphorylated, is repositioned within the cell, resulting in heightened vascular permeability [76]. Several other agents also contribute to this phosphorylation-mediated phenomenon. For example, the tripeptide proline-glycine-proline, originating from the breakdown of collagen, a common occurrence during tumor progression [77], is acetylated under normal physiological conditions. This tripeptide enhances the phosphorylation of VE-cadherin via the CXCR2 signaling pathway [78]. Similarly, angiomodulin, produced in bladder cancer cells and fibroblasts, interacts with integrin ανβ3, initiating the formation of actin stress fibers that lead to weakened intercellular connections mediated by VE-cadherin [79].

In addition, various cytokines are implicated. IL-1β, commonly found in diverse types of cancer, activates RhoA signaling pathways when it binds to IL-1R, which then results in the phosphorylation of VE-cadherin [80]. IL-8, associated with innate immune responses, binds to CXCR1 and CXCR2, activating VEGFR2 phosphorylation through Src, which in turn triggers RhoA activation and junctional protein reorganization [39].

Likewise, pancreatic adenocarcinoma-upregulated factor (PAUF) acts specifically by interacting with the CXCR4/TLR2 complex, thereby upregulating eNOS and activating Src to enhance VE-cadherin phosphorylation. Stem cell factor (SCF), which is ubiquitously expressed, interacts with the cKit receptor [81]. This results in cKit phosphorylation and subsequent NOS activation, producing nitric oxide (NO) that leads to S-nitrosylation of β-catenin and p120-catenin, culminating in their separation from VE-cadherin.

Other signaling molecules like Semaphorin 3A, often found in glioma stem-like cells, bind to the NPR1–plxA1 complex and activate Src, facilitating the internal relocation of VE-cadherin [82]. Finally, IL-6, produced predominantly in macrophages and very commonly also in cancer cells, binds to the IL-6R-gp130 heterodimer, activating STAT3. While STAT3 itself doesn’t directly regulate junctional proteins like ZO-1 and occludin, it does indirectly affect vascular permeability by upregulating VEGF expression [83].

### Factors causing endothelial cell contraction

In addition to affecting molecules that connect cells, certain elements also influence endothelial cells by causing them to contract and retract, which results in their reduced dimension (for a comprehensive review see here [27]. This action enlarges the space between adjacent cells, and amplifies the permeability of the barrier they form.

CCL2, generated by various cell types such as cancer cells, fibroblasts, and even endothelial cells, interacts with the CCR2 receptor to set off contractions in the actin-myosin complex [84].

Histamine, primarily originating from mast cells but released by several kinds of cancer cells [85], engages the H1R receptor, initiating actomyosin contractions through the RhoA-ROCK signaling pathway [86].

MASP-1 and thrombin work in conjunction with protease-activated receptor 1 (PAR1) to boost intracellular calcium levels. This elevated calcium concentration triggers the phosphorylation of myosin light chains. Subsequently, through the activation of the Rho-associated coiled-coil kinase (ROCK), actin fibers are reorganized. This leads to the retraction of proteins located at cellular junctions. Moreover, elevated levels of MASP-1 are correlated with poorer prognosis in the progression of cervical cancer [87, 88].

Platelet-Activating Factor (PAF) is upregulated and released by different kinds of cancer [89, 90]. PAF stimulates the production of nitric oxide (NO) via NO synthase activation. This NO modifies a specific cysteine in the vasodilator-stimulated phosphoprotein (VASP), a protein that regulates actin. Within endothelial cells, VASP is linked to various cellular structures, including actin stress fibers, adherens junctions, and tight junctions. The nitrosylation of VASP alters the cellular architecture, affecting its structure and function [91].

### Fusing with immune cells

Although the theory that suggests fusion between tumor cells and immune cells is quite old, it had not been adequately verified for a long time [92]. However, recent data have provided more convincing physiological evidence supporting this theory. Cell lines from colon cancer and melanoma, as well as tumor cells derived from patients, have been shown to be capable of fusing with macrophages. The hybrid cells produced maintain the expression of specific genes that are characteristic of macrophages and not present in tumor cells before fusion. An example is the receptor for colony-stimulating factor 1 (CSFR1) [93]. This receptor grants the hybrid cells the capability to migrate towards the CSF factor, exhibiting a distinct chemotactic mechanism. This would represent a clear advantage for metastasizing tumor cells [94]. Although the mechanism has not been completely clarified, it is clear that CSF1R is involved in tumor progression [95, 96]. Despite the scarcity of data currently available, the hypothesis remains particularly intriguing: that tumor cells, by fusing with immune cells, may acquire characteristics inherent to immune cells. These characteristics could be utilized to their advantage, potentially making the metastatic process more efficient.

## Conclusions and open questions

Extravasation stands as a critical bottleneck in the metastatic process, offering a potential target for halting the progression of cancer. There is a burgeoning body of evidence suggesting that cancer cells may either directly or indirectly co-opt factors known to trigger inflammation, utilizing them for invasive maneuvers into targeted tissues during metastasis. Despite comprehensive studies, the mechanisms that underlie the particular affinity some cancer cells have for specific organs remain largely mysterious. One intriguing hypothesis posits that distinct cancer cells, each bearing its own unique array of inflammatory markers, might have the ability to selectively ‘home in’ on particular tissues. This selectivity could be determined by the efficiency with which these factors can alter the permeability of the endothelial barriers in different organs. Additionally, the precise intracellular signaling pathways responsible for shaping these unique inflammatory profiles await further elucidation. Gaining a deeper understanding of these complex variables could unveil an array of promising targets for therapeutic intervention, aimed at mitigating the lethal progression of metastasis, a leading cause of mortality among patients afflicted with various forms of malignant cancer.
